# Psychological therapy for mood instability within bipolar spectrum disorder: a single-arm feasibility study of a dialectical behaviour therapy-informed approach

**DOI:** 10.1186/s40814-020-00586-1

**Published:** 2020-04-15

**Authors:** Kim Wright, Gemma Palmer, Mahmood Javaid, Mohammod Mostazir, Tom Lynch

**Affiliations:** 1 Department of Psychology, Washington Singer Labs., University of Exeter, Perry Road, Exeter, EX4 4QG UK; 2 Biomedical Informatics Hub, University of Exeter, Perry Road, Exeter, EX4 4QG UK; 3 College of Life and Environmental Sciences, University of Exeter, Perry Road, Exeter, EX4 4QG UK; 4 Department of Psychology, University of Southampton, 44 Highfield Campus, Southampton, UK

**Keywords:** Bipolar disorder, Cyclothymic disorder, Dialectical behaviour therapy

## Abstract

**Background:**

We sought to evaluate the acceptability of a psychological therapy programme (Therapy for Inter-episode Mood Variability in Bipolar Disorder (ThrIVe-B)) for individuals with ongoing bipolar mood instability and the feasibility and acceptability of potential trial procedures. We also evaluated the performance of clinical and process outcome measures and the extent to which the programme potentially represents a safe and effective intervention.

**Method:**

We conducted an open (uncontrolled) trial in which 12 individuals with a bipolar spectrum diagnosis commenced the ThrIVe-B programme after completing baseline assessments. The programme comprised 16 group skills training sessions plus individual sessions and a supporting smartphone application. Follow-up assessments were at therapy end-point and 6 months post-treatment.

**Results:**

Nine participants completed treatment. Ten provided end-of-treatment data; of these, nine were satisfied with treatment. Interviews with participants and clinicians indicated that the treatment was broadly feasible and acceptable, with suggestions for improvements to content, delivery and study procedures. Exploration of change in symptoms was consistent with the potential for the intervention to represent a safe and effective intervention.

**Conclusions:**

Conducting further evaluation of this approach in similar settings is likely to be feasible, whilst patient reports and the pattern of clinical change observed suggest this approach holds promise for this patient group. Future research should include more than one study site and a comparison arm to address additional uncertainties prior to a definitive trial.

**Trial registration:**

Trial Registration: ClinicalTrials.gov NCT02637401; registered 22.12.15 (retrospectively registered).

As well as experiencing episodes of depression and mania or hypomania, some individuals with bipolar disorder report more short-lived, or less intense, fluctuations in mood on an ongoing basis. Ongoing mood instability is also present in cyclothymic disorder, which by definition involves frequent, brief mood swings.

There is a clear need for effective interventions for this inter-episode mood instability (IMI). First, it is relatively common: the 12-month prevalence of cyclothymic disorder is around 1% [1], and whilst the prevalence of IMI within bipolar I and II disorder is rarely reported, one study found around half of participants in their sample with euthymic bipolar I or II disorder to show marked current mood instability [2]. Second, addressing IMI may represent an opportunity for secondary prevention and healthcare cost reduction as it is associated with risk of developing a personally and financially costly full depressive or manic episode [3]. Third, there is evidence that IMI itself is associated with significant distress and impairment in the form of increased psychiatric comorbidity (anxiety disorders, substance use [4]) and poorer functioning [5, 6].

Currently, there is no established pharmacological strategy for IMI; moreover, relatively few pharmacological treatment trials focus upon it as a feature of bipolar disorders (BDs) [7–9]. Existing recommended psychological therapies for bipolar disorder typically address depression or relapse risk rather than IMI, which is rarely measured in trials of these therapies. Whilst there are a small number of published studies investigating psychological therapies for cyclothymic disorder [10–12], none specifically addresses IMI across the full bipolar spectrum as reflected in both therapy design and inclusion criteria.

To address this treatment gap, we have taken an existing psychological therapy that addresses mood instability in personality disorders (dialectical behaviour therapy (DBT) [13]) and adapted it for IMI. DBT directly addresses “stable instability” of mood (whereby variable mood is the expected pattern) and contains both emotional acceptance techniques such as mindfulness and emotional change techniques. Nevertheless, some adaptations are likely to be necessary for clients with IMI: standard DBT targets key processes hypothesised to contribute to mood instability related to negative emotion (e.g. anger, sadness), but does not equip patients to manage hypomanic mood states. In addition, the primary outcomes in trials of standard DBT (mainly around self-injury) do not address key areas of concern for individuals with bipolar disorder, such as presence and persistence of depressive and manic states. This suggests that bespoke development and testing of this approach in this population is required. Several studies have examined modified versions of DBT as an intervention for bipolar disorder with encouraging results [14–17]; however, none has been fully powered randomised controlled trials in adults, none has specifically selected those with IMI and none has included individuals across the full bipolar spectrum. In response, we have developed a DBT-informed therapy programme adapted for IMI (the ThrIVe-B programme).

Prior to conducting a randomised pilot trial of ThrIVe-B, we wished to address uncertainties regarding the acceptability of the intervention and of key future trial procedures. This included recruitment of this patient group, who are not specifically identified by existing health care services. Case series evaluation is commonly used as a first step to address such uncertainties [18], as well as to conduct preliminary assessment of safety in a small number of individuals. Because our intervention is group-based and therefore does not lend itself to traditional case series methodology, we conducted an uncontrolled (open) feasibility study of two consecutive iterations of the programme. Our aims were to ascertain (i) the likely acceptability of the intervention to patients, to inform further treatment development; (ii) the feasibility and acceptability of study procedures, to inform a future randomised pilot trial; (iii) the extent to which any changes in clinical and process outcome measures are consistent with the intervention having potential as a safe and effective approach for this client group (assessed through pattern of improvement/deterioration in symptoms, including suicidality, and incidence of serious adverse events resulting from trial involvement). In addition, we sought to examine the performance of candidate outcome measures in terms of pre-post treatment correlations and potential sensitivity of the measures to detect change over the course of treatment.

## Method

### Trial design

Ours was an uncontrolled (open) trial with outcome evaluation points post-treatment and 6 months post-treatment. The study protocol is supplied as an additional file.

### Participants

Participants were required to be aged 18 or above and to have current bipolar mood instability, the definition of this being informed by DSM-V criteria for cyclothymic disorder (over the past 2 years numerous periods with hypomanic symptoms that do not meet criteria for a hypomanic episode and numerous periods with depressive symptoms that do not meet criteria for a depressive episode, continuing into the past month), and to be willing to engage in psychological therapy that focusses primarily on ongoing mood instability and its consequences. Because of the focus upon IMI as a trait that occurs across the bipolar spectrum, participants were required to meet DSM-IV [19] criteria for a bipolar spectrum condition (bipolar I disorder, bipolar II disorder, bipolar disorder not otherwise specified or cyclothymic disorder).

Patients were not able to participate in the study if they were currently experiencing mania or substance dependence disorder, were at high risk of attempting suicide (judged to be at immediate risk, according to the local research centre risk assessment tool), were currently receiving other psychological therapy, potentially placed other group members at significant risk (for example, through reported history of physical violence in similar settings) or lacked capacity to consent to research participation. Individuals presenting with another area of difficulty that the therapist and participant believed should be the primary focus of intervention (for example, prominent symptoms of post-traumatic stress disorder) were not included in the study, nor were individuals presenting with difficulties characteristic of borderline personality disorder rather than bipolar disorder (frequent and serious deliberate self-harm, marked disturbance in ability to form or maintain interpersonal relationships), as standard DBT is likely to be a more appropriate intervention for this group.

Potential participants were identified and approached by staff members in mental health assessment teams. Other than assessment, short-term support and medication advice, participants were not receiving ongoing secondary mental health care support.

As this was the first formal investigation of this subgroup, we did not pre-specify a target recruitment rate. Instead, we sought to use the findings to estimate recruitment rate for a subsequent controlled feasibility study. Informally, we considered that a recruitment rate of below two participants a month would be of concern for future studies because this would result in a long wait for participants to commence the group treatment.

We also surveyed clinicians from local mental health teams about their view of the therapy approach. A total of 10 clinicians participated in the survey, with four completing a brief semi-structured interview.

#### Intervention

The intervention consisted of 16 sessions of group skills training, supported by individual meetings of up to 30 min that occurred approximately monthly for each person over the 4 months of group sessions. In addition, there were two “booster” group sessions at 3 and 6 months post-intervention.

Content of group sessions was informed by DBT and followed a modular format: mindfulness I (2 sessions), emotion regulation (5), mindfulness II (2), distress tolerance (2), interpersonal effectiveness (4) and consolidation (1). Content was developed in consultation with individuals with personal experience of bipolar disorder and followed five key principles of DBT: (i) clearly structured treatment; (ii) application of behavioural therapy; (iii) emphasis on validation of emotional response; (iv) dialectical stance, balancing acceptance and change; and (v) integration of mindfulness practice [20]. Topics covered included skills for observing events, thoughts, emotions and bipolar symptoms without reacting impulsively, balancing lifestyle and activities to maximise mood stability and healthy rather than hypomanic positive mood, skills for downregulating emotion and negotiating interpersonal difficulties (which are often a consequence and a trigger of bipolar mood swings).

The group therapy sessions were delivered by two therapists. Individual sessions were with one of the two therapists, ensuring that all clients had the opportunity to meet separately with each therapist at least once during the programme. Therapy was delivered within a specialist psychological therapies service for adults with mood disorders.

In the current study, we ran the ThrIVe-B programme twice consecutively.

### Outcomes

#### Diagnostic evaluation

All participants were individuals diagnosed by their clinician as having (or probably having) bipolar or cyclothymic disorder. In addition, diagnosis was assessed by the research team using the structured clinical interview for DSM-IV [21], supervised by a clinical psychologist experienced in the use of the SCID.

#### Primary outcome measures: acceptability and feasibility

Acceptability of the therapy was measured in terms of (i) proportion of participants completing treatment (defined as attending at least 50% of the 16 group therapy sessions), (ii) participant ratings of treatment satisfaction, (iii) qualitative analysis of semi-structured interviews with participants at the end of therapy, (iv) clinician satisfaction ratings and (v) qualitative analysis of semi-structured interviews with clinicians. Overall participant satisfaction with the treatment was rated on a scale from 1 (very dissatisfied) to 4 (very satisfied); these data were collected as part of the standard service patient experience measure post-treatment. A more detailed measure of participant satisfaction was developed specifically for this study and asked participants, post-treatment and at follow-up, to rate their satisfaction on a scale from 1 (not at all) to 4 (very much so) with therapy content, group aspect, smartphone app, length of treatment, frequency of contact, fit with other commitments, discharge process and the ThrIVe-B programme. Neither measure has been externally validated.

Acceptability of the study procedures was addressed through qualitative analysis of the participant interviews and rates of completion of research measures. Feasibility was assessed via recruitment rate and qualitative analysis of interviews with clinicians.

#### Secondary outcome measures

##### Measures of symptoms

Manic symptoms were assessed using the 11-item, observer-rated Bech-Rafaelsen Mania Rating Scale (BMRS) [22] pre- and post-intervention. The BMRS has been shown to have adequate internal consistency and construct validity. The Altman Scale for Rating Mania (ASRM) [23], a five-item self-report measure of (hypo)manic symptom level, was completed on a session-by-session basis during the intervention: scores were used to inform and support therapy.

Depressive symptoms were measured using the nine-item Patient Health Questionnaire (PHQ9) [24], a widely used measure that can be used both to grade depressive symptom severity and establish probable presence of a major depressive episode pre- and post-intervention. The 21-item Beck Depression Inventory, second edition (BDI-II) [25] was also completed pre- and post-intervention, as well as on a session-by-session basis and at 6 months post-intervention.

Bipolar symptoms were also measured using the 16-item Internal State Scale (ISS) [26], which produces scores on four dimensions of depression, wellbeing, activation and perceived conflict.

Given high levels of anxiety amongst those with BD [27], the seven-item Generalised Anxiety Disorder Scale (GAD) [28] was used. As a general measure of psychiatric symptomatology, we included the 10-item Clinical Outcomes in Routine Evaluation Scale (CORE-10) [29].

##### Measures of sense of personal recovery and quality of life

The 36-item Bipolar Disorder Recovery Questionnaire (BDRQ) [30] was included as a measure of sense of personal recovery in people with bipolar disorder, as distinct from symptomatic or medical recovery. The BDRQ has been found to have adequate internal consistency and convergent validity. Finally, we included a measure of quality of life developed to be specific to individuals with bipolar disorder, the 12-item Brief Quality of Life in Bipolar Disorder scale (QoL.BD) [31].

##### Clinical process measures

Based upon the hypothesised core mechanisms of action of our intervention, we included measures of impulsive response to positive emotion (UPPS-P Impulsive Behaviour Scale [32]), avoidance behaviour (BADS: Behavioural Avoidance in Depression Scale [33]), mindfulness skills (KIMS: Kentucky Inventory of Mindfulness Skills [34]) and interpersonal functioning (IIP-32: Inventory of Interpersonal Problems—short version [35]). We also measured problematic beliefs about bipolar mood states (Brief-HAPPI: Brief Hypomanic Attitudes and Positive Predictions Inventory [36]) and a single-item asking about sense of fit with the group as social identification with treatment group may predict reduction of depression symptoms in individuals with unipolar depression [37]. Participants used a purpose-built smartphone application during part of the therapy within which they rated their mood at least once daily and viewed coping suggestions they had pre-entered. Findings pertaining to the application are not reported here.

##### Intervention and trial safety

This was assessed through examination of (i) rate of symptom deterioration (overall and with respect to suicidality) and (ii) rate of serious adverse events judged to be a consequence of the therapy or trial.

### Procedure

After giving written, informed consent to a member of the study team, participants completed a research interview including relevant sections of the SCID to determine study eligibility. Eligible participants wishing to continue then completed the acceptability, outcome and process measures. All participants used the monitoring app for 1 week prior to the first group therapy session. Participants returned completed BDI-II and ASRM measures to each group therapy session. Mid-way through the programme, participants completed the process measures.

Following the final group therapy session, participants completed a post-treatment interview including the SCID mood disorders section for the period since initial assessment, and the acceptability, outcome and process measures.

Six months after the end of the intervention phase, participants completed the SCID mood disorders section for the period since the previous assessment, the BDI-II and ASRM and measures of quality of life (QoL.BD), recovery (BDRQ) and acceptability of the intervention.

### Sample size

We aimed to recruit sufficient numbers for two iterations of the therapy programme, with each therapy group containing between 4 and 8 participants.

### Blinding

Because of the absence of a comparator arm, neither participants nor assessors were blind to participant allocation.

### Statistical methods

Descriptive statistics were used to summarise participant flow through the study and responses on quantitative measures of acceptability. To examine changes on clinical and process measures, means and standard deviations were calculated, as were effect sizes of change in scores from pre- to post-treatment [(μ2 − μ1)/pre-treatment SD]. For each participant, reliable change in their scores from pre- to post-treatment was calculated using the reliable change index (RCI: SEdiff *=* SD1√2 √1 − *r*) [38]. Analyses were per protocol (PP: including only those attending at least 50% of sessions) and intention to treat (ITT), using all data available, regardless of treatment received. Bivariate correlations were conducted to examine pre-post correlations within measures.

Qualitative data were subjected to thematic analysis. Transcripts from interviews with participants were coded by one rater, and a preliminary thematic framework developed. The transcripts were then coded by a second rater using the framework. Discrepancies in coding, and modifications to the framework, were discussed and a joint framework agreed. The transcripts were then recoded by rater 2 following the agreed framework.

Transcripts from interviews with staff were subjected to thematic analysis by a single rater and common themes identified.

## Results

### Recruitment and participant flow

Recruitment and data collection took place between January and July 2015 with follow-up ending in June 2016, when the final piece of data had been collected.

**Fig. 1:**
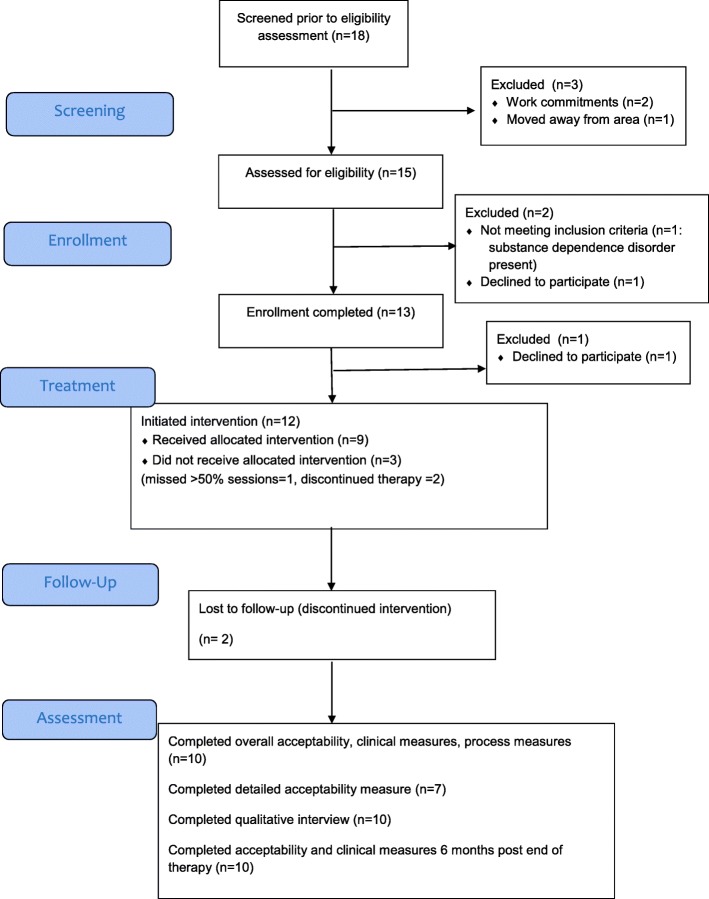
Study flow chart (adapted CONSORT diagram)

Eighteen individuals either contacted the study directly or gave consent for their clinician to pass their details to the research team. A total of 15 participants were assessed for eligibility; of these, three did not continue with the study, one person being ineligible to take part and two opting not to take part. Consequently, 12 people commenced the intervention, a rate of two participants entering the study per month and nine completed it (defined as attending at least 50% of group therapy sessions). Reasons for non-completion included change in work circumstances (1), not finding the approach to be helpful/being too unwell to attend (1) and unknown (1). The median number of group therapy sessions attended for those commencing treatment was 13/16 (range 3–16). Of the 12 individuals commencing treatment, 10 completed the research follow-up assessments at post-treatment and 6 months post-treatment. Recruitment and retention information is displayed in Fig. 1.

### Participant characteristics

Of the 12 individuals commencing therapy, 9 were female; the mean age of participants was 42 (SD 11). All participants were of white British ethnicity; the majority were married and in employment. Five were found to meet criteria for bipolar I disorder, five for bipolar II disorder and two for bipolar disorder not otherwise specified. The majority of participants were prescribed psychiatric medication. Table 1 gives details of participant characteristics.

**Table 1:** Baseline characteristics of the sample

**Variable**	**Number (%) / Mean (SD)**
Age (M (SD))	42 (11)
Gender	9 (75%) female
	3 (25%) male
Ethnicity	12 (100%) white British
Marital status	7 (58%) married
	5 (42%) single
Employment status	8 (67%) employed
	1 (8%) retired
	3 (25%) unemployed
Research diagnosis	5 (42%) bipolar I disorder
	5 (42%) bipolar II disorder
	2 (17%) bipolar disorder NOS
% prescribed mood stabilising medication	7 (58%)
% prescribed antidepressant medication	7 (58%)
% prescribed any psychiatric medication	8 (67%)

### Participant acceptability ratings

Nine out of 10 participants reported being mostly or very satisfied with treatment at post-treatment, with all reporting being at least slightly satisfied with the ThrIVe-B programme at 6 months follow-up. The areas associated with highest satisfaction were group-based nature of the treatment, length of treatment, frequency of contact with therapists and the referral and discharge process. The area associated with the lowest satisfaction was the use of the smartphone application. See Table 2 for acceptability ratings.

**Table 2:** Acceptability ratings post-treatment and at follow-up

**Item**	**Valid, *n***	**Range**	**Mean (SD)**	**Valid, *n***	**Range**	**Mean (SD)**
How satisfied were you with the type of treatment that you received?	10	1–4	3.2 (0.92)	–	–	–
The things we covered in this therapy programme have been helpful to me	7	2–4	2.86 (0.90)	10	2–4	3.20 (0.92)
I have been happy with the group-based nature of this therapy programme	7	1–4	3.00 (1.15)	9	2–4	3.44 (0.73)
I have been happy with the use of a smartphone application within this therapy programme	7	1–4	2.28 (1.38)	10	1–4	2.40 (1.07)
I am happy with the length of this treatment programme	7	2–4	3.00 (0.82)	10	2–4	3.30 (0.82)
I have been happy with the frequency of contact with therapists over the course of the therapy programme	7	3–4	3.85 (0.38)	10	3–4	3.60 (0.52)
It was easy to fit in this therapy programme alongside my commitments	7	1–4	2.71 (1.11)	10	1–4	2.90 (0.99)
I am happy with the process for referring me on or discharging me from the service	6	2–4	3.50 (0.84)	10	1–4	3.40 (0.97)
I am satisfied with the ThrIVe programme as a treatment	7	1–4	3.00 (1.15)	10	2–4	3.10 (0.99)

Items are rated from 1 (not at all) to 4 (very much so) other than the first item which is rated from 1 (very dissatisfied) to 4 (very satisfied). Due to an error, 3 participants completed overall acceptability rating post-treatment, but did not complete the detailed measure

### Qualitative information pertaining to acceptability

Three overarching themes were identified: *engagement* in therapy, *process* of therapy and *impact* of therapy. Each of these contained second and third-order subthemes (see Supplementary Material 1). The engagement theme included valued aspects of the therapy delivery and structure and potential areas for improvement. The aspects mentioned most frequently by participants as positive included the balance of individual and group sessions and the presence of individual contact. All participants gave opinions on the app, including suggestions for improvements in delivery and content. Within the process theme, all but one participant commented upon the benefit of being in a group of people with similar experiences, with individual participants also commenting on specific aspects of this such as learning from others, and the group itself being an incentive for attendance. Two participants voiced disadvantages of the group aspect of the therapy, namely feeling different and isolated from others and not wanting to hear about or share problems. With regard to therapy content, all modules were mentioned as helpful by at least one participant. As well as learning skills, learning more about oneself and having the space to reflect were seen as valuable. Where difficulty or dissatisfaction the content of the therapy was voiced, this was in terms of knowing the concepts, but it being hard to apply in practice/in the moment, techniques being less effective in severe mood states, already using some of the ideas before coming to the group and a sense that mood swings are not within personal control.

In terms of the impact of therapy, the most commonly cited changes in intrapersonal behaviour included increased acceptance of the self, internal events and external events; ability to step back from emotions and situations; ability to make better choices (rather than acting impulsively); and being able to let go of experiences. The ability to observe others and see things from their perspective was the most commonly endorsed aspect of interpersonal behavioural change. Participants who commented upon impact on symptoms reported symptoms persisting, but also symptom reduction and stabilisation of mood. It is notable that some participants endorsed both persistence of symptoms and improvement or stabilisation. Four participants commented upon a sense that therapy had prevented symptoms worsening. Impact on relationships was described by participants as including positive effects of change in themselves on others around them and a sense of building better relationships. Some participants also commented that therapy impacted positively upon their functioning at work.

### Referrer and clinician feedback

All 10 staff considered the group format and length of treatment acceptable and appropriately placed in the care pathway and viewed DBT as an appropriate intervention for this population. The use of the smartphone application in the group was viewed least positively of all aspects of the intervention. Overall, 9/10 clinicians were “very much” satisfied with the programme as a treatment option for their clients, and one “moderately”.

Common themes identified from the semi-structured interviews were a distinction between different types of BD and the treatments that best suit each type, the state of current provision for this patient group in services, the suitability of a group intervention for people with BD and specific comments and recommendations for ThrIVe-B groups. In summary, clinicians described the ThrIVe-B approach as likely to be appropriate for people with bipolar spectrum conditions who sought psychological and functional support in the community or for those with cyclothymic disorder and less appropriate for those tending to become very unwell very quickly, resulting in hospital admission. Clinicians spoke of the limitations of the current provision, acknowledging that people with BD are often excluded from primary care psychological therapies and that those who do receive a service from secondary care are more likely to be offered care coordination rather than psychological intervention. They all spoke positively about the benefits of providing psychological interventions in a group environment; however, they noted this would not suit all participants.

### Study and therapy safety

There was one serious adverse event (a participant tried to end their life) 4 months after the end of the treatment phase (2 months before the final follow-up point): this was judged not to be a result of the therapy or the trial procedures.

As another means of assessing safety of the intervention, we explored change in self-reported frequency of suicidal ideation (score on item 9 of PHQ9) from baseline to end of treatment. In no participants did this increase: three participants reported no ideation at baseline and this remained the case post-treatment; in two participants, ideation was reported at baseline and did not change; five participants reported ideation at baseline but not at the end of treatment.

### Secondary outcome measures

#### Symptoms, sense of personal recovery and quality of life

Mean scores on the clinical outcome measures at baseline, post-therapy and at 6 months post-end of intervention are given in Table 3. Also, displayed are pre-post correlations for each measure and the number of participants showing reliable improvement, reliable deterioration and no change upon each clinical outcome measure for both per protocol and ITT analyses.

**Table 3:** Mean scores and change on clinical outcome measures at baseline and follow-up assessments

**Variable**	**Baseline, mean (SD)** **whole sample**		**Pre-treatment, mean (SD)** **ITT/PP sample**	**Post-treatment, mean (SD)**	**Pre-post correlation (*R*)**	**Effect size (*d*)**	**Reliable change (deterioration, no change, improvement)**	**Six months post-treatment follow-up, mean (SD)**
BDI-II	32.75 (11.66)	ITT	30.30 (11.23)	15.60 (10.81)	0.42	1.31	0, 3, 7	21.80 (13.03)
		PP	29.67 (11.72)	13.00 (7.45)	0.44	1.42	0, 2, 7	19.78 (12.04)
PHQ9	18.25 (6.34)	ITT	17.50 (6.67)	10.00 (7.09)	0.64	1.12	0, 4, 6	–
		PP	16.78 (6.65)	8.56 (5.75)	0.59	1.24	0, 3, 6	
GAD7	13.92 (5.47)	ITT	12.90 (5.43)	4.90 (3.18)	0.11	1.47	0, 2, 8	–
		PP	13.11 (5.71)	4.89 (3.37)	0.11	1.44	0, 2, 7	
BMRS	4.08 (6.40)	ITT	2.40 (2.59)	1.70 (2.54)	0.97	0.27	0, 10, 0	–
		PP	2.56 (2.70)	1.89 (2.62)	0.97	0.25	0, 9, 0	
ASRM^1^	3.75 (4.39)	ITT	4.40 (4.55)	5.30 (2.11)	0.36	0.20	2, 6, 2	6.00 (4.27)
		PP	4.67 (4.74)	5.67 (1.87)	0.31	0.21	2, 5, 2	6.33 (4.39)
CORE-10	20.08 (6.20)	ITT	19.80 (6.71)	12.30 (7.39)	0.30	1.12	1, 3, 6	–
		PP	19.33 (6.95)	10.89 (6.25)	0.21	1.22	1, 3, 5	
QoL.BD^2^	28.91 (8.35)	ITT	30.00 (6.61)	38.67 (7.86)	0.23	1.31	0, 5, 4	34.40 (10.50)
		PP	30.50 (6.89)	40.25 (6.69)	0.12	1.42	0, 4, 4	35.44 (10.57)
BDRQ	1877.63 (255.25)	ITT	1910.40 (268.54)	2179.39 (489.33)	0.52	1.00	1, 4, 5	1994.58 (230.13)
		PP	1951.14 (249.92)	2240.02 (477.51)	0.55	0.81	1, 4, 4	2022.77 (225.03)
ISS Activation	210.83 (150.12)	ITT	236.00 (148.56)	179.00 (144.03)	0.57	0.38	0, 8, 2	–
		PP	252.22 (147.88)	192.22 (146.18)	0.54	0.54	0, 7, 2	
ISS Conflict	205.00 (133.52)	ITT	198.00 (128.31)	114.00 (72.60)	0.78	0.65	0, 7, 3	–
		PP	195.56 (135.84)	115.56 (76.83)	0.79	0.59	0, 6, 3	
ISS Wellbeing	98.33 (67.53)	ITT	108.00 (70.36)	144.00 (87.71)	0.82	0.51	0, 8, 2	–
		PP	118.89 (65.09)	157.78 (80.74)	0.74	0.60	0, 7, 2	
ISS Depression	109.17 (52.99)	ITT	96.00 (47.19)	66.00 (67.36)	0.57	0.64	1, 5, 4	–
		PP	90.00 (45.83)	51.11 (51.10)	0.35	0.85	0, 5, 4	

Nine participants completed 6 months post-treatment follow-up

*ITT* intention-to-treat sample (*n* = 12), *PP* per protocol sample (*n* = 9), *BDI-II* Beck Depression Inventory—second edition, *PHQ9* Patient Health Questionnaire, *GAD7* Generalised Anxiety Disorder Scale, *BMRS* Bech-Rafaelson Mania Rating Scale, *ASRM* Altman Scale for Rating Mania, *CORE-10* Clinical Outcomes in Routine Evaluation Scale, *QoL.BD* Brief Quality of Life in Bipolar Disorder Scale, *BDRQ* Bipolar Disorder Recovery Questionnaire, *ISS* Internal State Scale

^1^Completed prior to session 1 of therapy

^2^ITT *n* = 11; PP *n* = 8

As can be seen, mean levels of anxiety and depression reported by participants were clinically significant at baseline, with mean BDI-II score in the severe range, mean PHQ9 score moderately severe, mean GAD7 score moderate and general distress and impairment (as measured by the CORE-10) in the moderate to severe range. In contrast, mean BRMS and ASRM scores fell in the non-clinical range.

Mean score on the QoL-BD at baseline fell within one standard deviation of mean score on this measure in a sample of individuals with bipolar disorder prior to commencing a psychoeducation intervention (35.356 [SD 10.10]) [39], whilst mean score on the BDRQ (sense of personal recovery) fell more than one standard deviation below mean score in a sample of individuals with bipolar disorder who took part in the original measure validation study (2357.7 [SD 414.0]: Jones et al. [30]).

As a means of assessing the possible sensitivity of the outcome measures to detect change in post-treatment, we used the RCI. This indicated reliable improvement in at least half of the participants on the following measures: BDI-II, PHQ9, GAD7, CORE, BDRQ (ITT analyses) and QoL-BD (PP analysis only). Without a comparison group, it is not possible to infer any effect of treatment upon scores on these measures; however, the presence of reliable change in at least half of the sample is consistent with the possibility that these measures could be sensitive to treatment effects.

In contrast, for the majority of participants, no reliable change was observed in scores on the Bech Mania Scale, ASRM and ISS.

Use of the RCI and consideration of effect size also allowed us to explore the extent to which patient outcomes were consistent with this being a safe and effective treatment. In almost all participants where change was observed, this was in the direction of reliable improvement. Whilst the current study design cannot test efficacy, this finding supports a case for further investigation. Reliable deterioration was observed on only three measures: the ASRM (*n* = 2), BDRQ (*n* = 1) and CORE-OM (*n* = 1). That this pattern was not observed across their scores on other measures of symptoms and quality of life suggests that there was not a subgroup of individuals upon which the treatment may have had a marked deleterious effect.

In SCID interviews conducted at the end of treatment, 5/10 participants reported at least one episode of depression and 5/10 reported at least one episode of hypomania since the baseline assessment, with 7/10 reporting at least one episode of any type. Six months after the end of treatment, 5/10 reported depression and 2/10 hypomania, with 7/10 reporting at least one episode of any type. No participants reported experiencing mania over the course of the study.

#### Clinical process measures

Table 4 gives mean scores on process measures at baseline, midway through therapy and post-treatment, as well as the results of reliable change analyses on per protocol data. Reliable change was observed in at least half of participants on the following measures: HAPPI, UPPS-P NU and UPPS-P PU, and for all but one data point, change was in the direction of reliable improvement. Again, effect of treatment upon these scores cannot be inferred; nevertheless, the presence of reliable change in at least half of the sample on these measures is consistent with the possibility that these measures could be sensitive to treatment effects.

**Table 4:** Mean scores and change on process measures at baseline and follow-up assessments (PP sample)

	**Pre-treatment mean (SD)**	**Post-treatment mean (SD)**	**Pre-post correlation (*R*)**	**Effect size (*d*)**	**Reliable change (deterioration, no change, improvement)**
BADS total	71.44 (27.51)	100.00 (20.35)	0.37	1.04	0, 5, 4
KIMS_OB^1^	40.17 (9.68)	44.33 (7.53)	0.64	0.43	0, 5, 1
KIMS_DES^1^	26.33 (6.86)	28.50 (3.15)	0.65	0.32	0, 4, 2
KIMS_AW^1^	23.67 (8.59)	29.33 (8.16)	0.59	0.66	0, 4, 2
KIMS_AC^1^	21.50 (8.98)	25.67 (5.20)	0.23	0.46	0, 4, 2
HAPPI	2333.25 (821.02)	1497.94 (760.96)	0.25	1.02	1, 2, 6
IIP (*n* = 8)	58.44 (18.37)	49.75 (18.27)	0.56	0.51	0, 6, 2
UPPS-P NU^2^	38.88 (5.84)	34.44 (8.31)	0.22	1.05	0, 4, 4
UPPS-P PU^2^	45.88 (9.71)	37.56 (11.40)	0.04	0.85	0, 3, 5
UPPS-P PM^2^	25.99 (6.22)	24.78 (5.19)	0.53	0.20	1, 7, 0
UPPS-P SS^2^	30.88 (6.45)	30.22 (7.26)	0.66	0.27	0, 7, 1
UPPS-P PR^2^	23.00 (6.76)	22.11 (4.96)	0.57	0.09	1, 6, 1
Group fit	2.38 (1.06)	3.78 (1.09)	− .11	1.32	NA

*n* = 9 unless otherwise stated

*BADS* Behavioural Avoidance in Depression Scale, *KIMS* Kentucky Inventory of Mindfulness Skills, *HAPPI* Brief Hypomanic Attitudes and Positive Predictions Inventory, *IIP-32* Inventory of Interpersonal Problems—short version, *UPPS-P* UPPS-P Impulsive Behaviour Scale

^1^*n* = 6 completed post-treatment KIMS

^2^*n* = 8 completed both time points

## Discussion

This open feasibility trial represents the first attempt to evaluate the potential of a DBT-informed approach for individuals with bipolar mood instability occurring outside of full affective episodes. We sought to evaluate the feasibility of recruiting participants to a trial of this nature and the acceptability of the programme to those who were offered it.

Conducting further evaluation of this approach appears feasible (see Supplementary Material 2 for a summary of findings). Given that recruitment was from only a small number of mental health assessment teams over a 6-month period, the rate of participants consented and eligible for the trial per fortnight is commensurate with the feasibility of a larger trial, using more diverse recruitment sources. A recruitment rate of at least 2 participants per month in a future feasibility randomised controlled trial would allow two study sites to recruit 50 participants in just over a year, adequate for feasibility assessment; this trial could examine the impact of using additional referral sources (such as self-referral and primary care) in preparation for a definitive trial. It also speaks to bipolar mood instability being an aspect of BD that patients and clinicians believe deserves specific attention and treatment. Furthermore, clinician feedback supports the acceptability of this approach to potential referrers, and hence the feasibility of future recruitment from mental health teams. In terms of follow-up data collection, 83% of participants starting treatment returned follow-up data at post-treatment and 6 months post-treatment, suggesting that completeness of follow-up data in future trials is likely to be at an acceptable level.

Overall, it appears that participants found the treatment approach acceptable, with 75% of participants completing treatment and only one participant dissatisfied at the end of the therapy period, with none dissatisfied at 6 months post-treatment. Further work is required to determine what should constitute “treatment completion” in terms of session attendance and the rate of therapy completion that renders the intervention viable from a health economics perspective. Examination of the themes arising from in-depth interviews with participants supported the value of the mode of delivery and the therapy content, whilst some themes indicated areas for improvement, for example, modifications to the smartphone app to increase ease of use.

We also sought to examine the extent to which any changes in outcome and process measures are consistent with the intervention having potential as a safe and effective approach for this client group. There was no evidence for any participant showing a consistent pattern of reliable deterioration in their scores on the outcome measures, and it does not appear that the intervention was associated with increased suicidal thinking. It should be noted though that two participants did not complete the end of therapy outcome measures and therefore their possible response to treatment is unknown, although qualitative feedback was provided by one of these participants upon exiting therapy, and this indicated a positive view of the sessions attended and their effects. The current design is not able to test the efficacy of the intervention. Nonetheless, the finding that, on measures of depression, anxiety, general psychiatric symptoms and sense of personal recovery, at least half of participants showed reliable improvement suggests future testing of potential clinical benefit is indicated.

Finally, we sought to examine the performance of candidate outcome and process measures. Where reliable change in a substantial number of individuals was observed, this is consistent with these measures being sensitive to treatment effects and supports their use in future testing of this approach. No or little reliable change was observed on measures of mania symptoms and the Internal State Scale: it is not clear whether this is a consequence of measure insensitivity or due to a genuine absence of change. It is notable that mean levels of mania symptoms in the sample were in the non-clinical range at baseline, which have restricted the potential for decrease on these measures. Amongst the candidate process measures, those addressing impulsive response to affect (UPPS-P positive and negative urgency scales, and to some extent the BADS) and potentially unhelpful beliefs about mood states showed the greatest degree of reliable change across participants, supportive of their use in future evaluations of this approach.

Although commensurate with early-stage testing of a psychological intervention in a little-researched population, our relatively small sample size and the use of a single location and service in the delivery of the intervention limit the generalisability of our findings. Also, data on the typical patient population of the referring services was not available, limiting our ability to compare our sample with the service population. Including a comparison condition in further feasibility testing with a larger sample will be necessary in order to pilot the likely design of a future definitive trial. In addition, this will permit blinding of assessors thus reducing potential for bias. Furthermore, the current study did not select participants on the basis of a severity threshold with regard to IMI: future research should seek to incorporate dimensional measurement of IMI to permit this.

In conclusion, the ThrIVe-B programme appears to be broadly acceptable to both patients and their clinical teams, with several aspects that could be improved. Conducting further evaluation of this approach is likely to be feasible. Following modification to the therapy programme in line with the findings of the current study, future research should seek to evaluate the feasibility of a randomised controlled evaluation of the ThrIVe-B programme.

## Supplementary information

**Additional file 1.** Supplementary Material 1. Summary of thematic analysis of qualitative interviews with participants. Table reporting themes, sub-themes and exemplar quotations from thematic analysis of qualitative interviews with participants.

**Additional file 2.** Supplementary Material 2. Summary of Findings in Relation to Feasibility Aims. Table study findings in relation to each feasibility aim.

**Additional file 3.** Protocol. Group Dialectical Behavioural Therapy for Mood Instability within Bipolar Disorder: An Open Trial. Study Protocol. Study protocol.

## Data Availability

The datasets generated and analysed during the current study are not publicly available to protect participant confidentiality, but are available from the corresponding author on reasonable request, subject to any applicable regulatory approvals for secondary use of the data.

## Abbreviations

App: Smartphone application
ASRM: Altman Scale for Rating Mania
BADS: Behavioural Avoidance in Depression Scale
BD: Bipolar disorders
BDI: Beck Depression Inventory
BDRQ: Bipolar Disorder Recovery Questionnaire
IMI: Inter-episode mood instability
DBT: Dialectical behaviour therapy
DSM-V: Diagnostic and Statistical Manual of Mental Disorders, fifth edition
GAD7: Generalised Anxiety Disorder 7-item scale
ITT: Intention to treat
KIMS: Kentucky Inventory of Mindfulness Skills
PHQ9: Patient Health Questionnaire 9-item scale
QoL-BD: Quality of Life in Bipolar Disorder scale
ThrIVe-B: Therapy for Inter-episode mood Variability in Bipolar
UPPS-P: Positive and Negative Urgency, Premeditation, Perseverance, Sensation-Seeking scales

## References

[1] Faravelli C, Incerpi G (1985). Epidemiology of affective disorders in Florence: preliminary results. Acta Psychiatr Scand.

[2] Bonsall MB, Wallace-Hadrill SM, Geddes JR, Goodwin GM, Holmes EA (2012). Nonlinear time-series approaches in characterizing mood stability and mood instability in bipolar disorder. Proc R Soc B.

[3] Akiskal HS, Djenderedjian AH, Rosenthal RH, Khani MK (1977). Cyclothymic disorder: validating criteria for inclusion in the bipolar affective group. Am J Psychiatry.

[4] Henry C, Van den Bulke D, Bellivier F, Roy I, Swendsen J, M'Baïlara K, Siever LJ, Leboyer M (2008). Affective lability and affect intensity as core dimensions of bipolar disorders during euthymic period. Psychiatry Res.

[5] Gershon A, Eidelman P (2015). Inter-episode affective intensity and instability: predictors of depression and functional impairment in bipolar disorder. J Behav Ther Exp Psychiatry.

[6] Strejilevich SA, Martino DJ, Murru A, Teitelbaum J, Fassi G, Marengo E, Igoa A, Colom F (2013). Mood instability and functional recovery in bipolar disorders. Acta Psychiatr Scand.

[7] Fountoulakis KN, Kontis D, Gonda X, Yatham LN (2013). A systematic review of the evidence on the treatment of rapid cycling bipolar disorder. Bipolar Disord.

[8] NICE (2014). Bipolar disorder: assessment and management. NICE clinical guideline 185. Department of Health.

[9] Perugi G, Hantouche E, Vannucchi G, Pinto O (2015). Cyclothymia reloaded: a reappraisal of the most misconceived affective disorder. J Affect Disord.

[10] Fava GA, Rafanelli C, Tomba E, Guidi J, Grandi S (2011). The sequential combination of cognitive behavioral treatment and well-being therapy in cyclothymic disorder. Psychother Psychosom.

[11] Totterdell P, Kellett S (2008). Restructuring mood in cyclothymia using cognitive behavior therapy: an intensive time-sampling study. J Clin Psychol.

[12] Totterdell P, Kellett S, Mansell W (2012). Cognitive behavioural therapy for cyclothymia: cognitive regulatory control as a mediator of mood change. Behav Cog Psychother.

[13] Linehan MM. Skills training manual for treating borderline personality disorder. New York: Guilford Press; 1993.

[14] Eisner L, Eddie D, Harley R, Jacobo M, Nierenberg AA, Deckersbach T (2017). Dialectical behavior therapy group skills training for bipolar disorder. Behav Ther.

[15] Goldstein TR, Axelson DA, Birmaher B, Brent DA (2007). Dialectical behavior therapy for adolescents with bipolar disorder: a 1-year open trial. J Am Acad Child Adolesc Psychiatry.

[16] Goldstein TR, Fersch-Podrat RK, Rivera M, Axelson DA, Merranko J, Yu H, Brent DA, Birmaher B (2015). Dialectical behavior therapy for adolescents with bipolar disorder: results from a pilot randomized trial. J Child Adolesc Psychopharmacol.

[17] Van Dijk S, Jeffrey J, Katz MR (2013). A randomized, controlled, pilot study of dialectical behavior therapy skills in a psychoeducational group for individuals with bipolar disorder. J Affect Disord.

[18] Chan K, Bhandari M (2011). Three-minute critical appraisal of a case series article. Indian J Orthop.

[19] American Psychiatric Association (1994). Diagnostic and statistical manual of mental health disorders: DSM-IV.

[20] Koerner K. Doing dialectical behavior therapy: a practical guide. New York: Guilford Press; 2012.

[21] First MB, Spitzer RL, Gibbon M, Williams JB (2002). Structured clinical interview for DSM-IV-TR axis I disorders, research version, patient edition. SCID-I/P.

[22] Bech P, Rafaelsen OJ, Kramp P, Bolwig TG. The mania rating scale: scale construction and inter-observer agreement. Neuropharmacology. 1978;17:430-1. 10.1016/0028-3908(78)90022-9 673161

[23] Altman EG, Hedeker D, Peterson JL, Davis JM (1997). The Altman self-rating mania scale. Biol Psychiatry.

[24] Spitzer RL, Kroenke K, Williams JB (1999). Patient Health Questionnaire Primary Care Study Group. Validation and utility of a self-report version of PRIME-MD: the PHQ primary care study. JAMA.

[25] Beck AT, Steer RA, Brown GK (1996). Beck depression inventory-II. San Antonio.

[26] Bauer MS, Crits-Christoph P, Ball WA, Dewees E, McAllister T, Alahi P, Cacciola J, Whybrow PC (1991). Independent assessment of manic and depressive symptoms by self-rating: scale characteristics and implications for the study of mania. Arch Gen Psychiatry.

[27] Keller MB (2006). Prevalence and impact of comorbid anxiety and bipolar disorder. J Clin Psychiatry.

[28] Spitzer RL, Kroenke K, Williams JB, Löwe B (2006). A brief measure for assessing generalized anxiety disorder: the GAD-7. Arch Intern Med.

[29] Evans JM-C, Margison F, Barkham M, Audin K, Connell J, McGrath G (2000). C. CORE: clinical outcomes in routine evaluation. J Mental Health.

[30] Jones S, Mulligan LD, Higginson S, Dunn G, Morrison AP (2013). The bipolar recovery questionnaire: psychometric properties of a quantitative measure of recovery experiences in bipolar disorder. J Affect Disord.

[31] Michalak EE, Murray G, CREST. BD (2010). Development of the QoL. BD: a disorder-specific scale to assess quality of life in bipolar disorder. Bipolar Disord.

[32] Lynam DR, Smith GT, Whiteside SP, Cyders MA (2006). The UPPS-P: assessing five personality pathways to impulsive behavior.

[33] Kanter JW, Mulick PS, Busch AM, Berlin KS, Martell CR (2007). The Behavioral Activation for Depression Scale (BADS): psychometric properties and factor structure. J Psychopath Behav Assessment.

[34] Baer RA, Smith GT, Allen KB (2004). Assessment of mindfulness by self-report: the Kentucky Inventory of Mindfulness Skills. Assessment.

[35] Barkham M, Hardy GE, Startup M (1996). The IIP-32: A short version of the Inventory of Interpersonal Problems. Br J Clin Psychol.

[36] Mansell W, Jones SH (2006). The Brief-HAPPI: A questionnaire to assess cognitions that distinguish between individuals with a diagnosis of bipolar disorder and non-clinical controls. J Affect Disord.

[37] Cruwys T, Haslam SA, Dingle GA, Jetten J, Hornsey MJ, Chong ED, Oei TP (2014). Feeling connected again: interventions that increase social identification reduce depression symptoms in community and clinical settings. J Affect Disord.

[38] Christensen L (1986). A method of assessing change in a single subject: an alteration of the RC index. Behav Ther.

[39] Todd NJ, Jones SH, Hart A, Lobban FA (2014). A web-based self-management intervention for bipolar disorder ‘living with bipolar’: a feasibility randomised controlled trial. J Affect Disord.

