# Supplementary material for: Psychological therapy for mood instability within bipolar spectrum disorder: a single-arm feasibility study of a dialectical behaviour therapy-informed approach
Source: Pilot Feasibility Stud. 2020 Apr 15;6:46. doi: 10.1186/s40814-020-00586-1 (PMC7158125; doi:10.1186/s40814-020-00586-1)
Supplement: Supplementary file 1 — Additional file 1. Supplementary Material 1. Summary of thematic analysis of qualitative interviews with participants. Table reporting themes, sub-themes and exemplar quotations from thematic analysis of qualitative interviews with participants. [file 40814_2020_586_MOESM1_ESM.docx]

**Supplementary Material 1: Summary of thematic analysis of qualitative interviews with participants**

| Theme | Subtheme | Exemplar Quote |
| --- | --- | --- |
| 1. Engagement   1.1. Delivery Mode (n = 11) | Balance of group and individual sessions (n = 5) | (Participant 10)  It was great to have the combination and the balance of being able to have a one to one every two weeks…  I really appreciated having the mix of group and individual time. |
|  | Suggestions for individual sessions (n = 2) | (Participant 5)  If you know the time of the session that it helpful, rather than staying or not after the group. |
|  | App Suggestions/ negative affective response/ difficulties **practical** (n = 10) | (Participant 10)  When high I forgot to do it. |
|  | App suggestions/ room for improvement **content** (n = 10) | (Participant 1)  It would be useful in the app to be able to access techniques when you want them. |
|  | Suggestions for group exercises (including “Didn’t like role plays”) (n = 3) | (Participant 3)  I didn’t like the role plays. Better to give people the option so they don’t have to do it. |
|  | Repetition (n = 1) | (Participant 3)  Repetition (of ideas was helpful) |
|  | Benefit of individual sessions (n = 10) | (Participant 10)  Having the individual time was an opportunity to talk and receive feedback on personal issues that I wanted to discuss in more depth than in group time. |
|  | Manner of delivery (n = 2) | (Participant 8)  If I had read the content in isolation I would have said “what’s this new age mumbo jumbo?” but the way it was put together, with enthusiasm, and encouragement of our involvement. If it was just lectured I may not have picked it up. |
|  | Appreciated not being questioned directly (n = 1) | (Participant 9)  Not being questioned directly. |
|  | Able to complete homework (right amount of homework) (n = 2) | (Participant 8)  I never found it too much of a problem (the homework) because if I did it daily it didn’t take too long |
|  | Materials helpful / clear (n = 3) | (Participant 10)  Since the course ended I have been struggling with depression and found that the course notes and things I learned from the thrive programme have been of significant help in helping to lift my mood. |
|  | Liked small group size (n = 3) | (Participant 9)  I liked the small group size. |
| 1.2. Relationship to Research (n = 2) | Positive view of taking part in research (n = 1) | (Participant 2)  I like research |
|  | Viewed it as research rather than treatment (n = 2) | (Participant 6)  Did not see it as a treatment – you were researching me. |
| 1.3. Practicalities (n = 9) | Life gets in the way (n = 1) | (Participant 3)  Life events sometimes made it difficult to go. |
|  | Timing (n = 5) | (Participant 4)  It was difficult to get the time off. Evening would have been easier. |
|  | Environment (n = 2) | (Participant 4)  I liked it, liked the room, looking at the fountain. |
|  | Cost of attending (n = 1) | (Participant 1)  The cost of parking and petrol. Opportunity cost for work. |
|  | Motivated to attend (i.e. by commitment they have made and the opportunity given to them) (n = 2) | (Participant 9)  I had made a commitment at the start in the assessment session. I was given an opportunity and I wanted to take it. |
|  | Helpful to have structure (of regular group session) (n = 3) | (Participant 7)  The discipline of going to the group was helpful, got me out. I liked the structure. |
| 1.4. Affective response to treatment (n = 6) | Enjoyed it (n = 3) | (Participant 2)  I really enjoyed it. I never felt unenthusiastic about coming. |
|  | Uncomfortable (n = 1) | (Participant 6)  Uncomfortable at times. |
|  | Initially anxious about group (n = 3) | (Participant 11)  I was nervous to start with, anxious about being in a group. |
|  | Exciting (n = 1) | (Participant 9)  I could identify with what other members were saying – this was exciting at times. |
|  | Could not get really involved in it (n = 1) | (Participant 6) I could not get really involved in it |
| 1.5. Beliefs about treatment (n = 1) | Do not believe I can influence my symptoms (n = 1) | (Participant 7)  I don’t have the control. |
| 1. Process   2.1. Group Factors (n = 10) | Learning from others (n = 2) | (Participant 8)  the group dynamic meant that answers to questions bounced off different people |
|  | Group pleasant to be in (n = 2) | (Participant 8)  A good group of people |
|  | Being with/ sharing/ hearing from people with similar experiences/ identifying with others (n = 9) | (Participant 3)  It gave me a measure (against others) of how I am doing ok – hearing their stories |
|  | Others modelling techniques (n = 1) | (Participant 5)  Listening to others. It made it more understandable, helps to embrace mindfulness as others are trying it. |
|  | Don’t want to talk about my problems with others or hear about theirs (n = 1) | (Participant 1)  I am not interested in group therapy. I don’t want to hear about other people’s issues and talk about mine. |
|  | Group factors kept me coming back (n = 1) | (Participant 9)  I liked hearing others’ experiences – it kept me coming back |
|  | Felt different from others/ isolated (n = 1) | (Participant 6)  What I have learned the most is how distant I am from other people. |
| 2.2. Most useful content (n = 10)  Modules found helpful: | Chance to learn strategies and techniques (n = 2) | (Participant 4)  There were good practical tips throughout the whole course |
|  | Pick which are helpful (n = 1) | (Participant 3)  Some things fitted, others didn’t. |
|  | Mindfulness skills (n = 3) | (Participant 11)  I liked the mindfulness at the start, one mindfully, the practical meditation stuff. |
|  | Emotion regulation techniques (n = 2) | (Participant 4)  Emotion regulation framework – does my mood fit the situation? Really useful. |
|  | Distress tolerance techniques (n = 1) | (Participant 4)  Distress tolerance. Accepting I can have control over how I feel and that I can control how I feel but not how others feel. |
|  | Interpersonal skills (n = 2) | (Participant 5)  DEAR MAN – dealing with interpersonal situations. I am not sure if I executed it well but now I know it. I think about it quite a lot – it’s a valuable tool. |
|  | Looking after myself (master skills) (n = 1) | (Participant 9)  Looking after myself (master skills) |
|  | Relaxation element (n = 2) | (Participant 7)  Encouraging myself into good moods and taking the tension away. I recognised this and know it works |
|  | Balancing activities (n = 1) | (Participant 9)  Balance of activities across the week |
|  | Awareness of moods (n = 2) | (Participant 11)  Being more aware of my mood and using some of the skills on the course (has helped), e.g. stopping and meditation in high mood. |
|  | Emergency box (n = 1) | (Participant 9)  Emergency box (was helpful) |
|  | Idea of using/taking small steps (n = 1) | (Participant 5)  The small steps idea (was helpful) |
|  | Learning more about self (n = 4) | (Participant 6)  What I have learned the most is how distant I am from other people |
|  | Having the space and opportunity to reflect/ being more reflective (n = 3) | (Participant 11)  thinking about it after, I realised I’m not very good at some of that stuff |
|  | Difficult to do mindfulness (n = 1) | (Participant 6)  Mindfulness – I am not too good at breathing and relaxing. |
|  | Doing skills already (n = 2) | (Participant 1)  I have already trained myself not to do that (ruminate). |
|  | Positive view of app (n = 3) | (Participant 3)  The app in theory is brilliant, |
| 1. Impact   3.1. Changes in intrapersonal behaviour (n = 8) | Stepping back, taking a moment (n = 3) | (Participant 11)  I am slowing down and thinking before acting. Before a conversation |
|  | Acceptance of self, moods, thoughts and of external factors (n = 4) | (Participant 11)  I accept the fact that my mind will have thoughts coming in and that’s ok…  I accept that I will have low and high moods – it’s part of being human. |
|  | Making better choices / being less impulsive (n = 5) | (Participant 2)  But I manage it (depression) better and make better choices…  Before I was usually caught up in the moment. Now I think about consequences. |
|  | Letting go (n = 3) | (Participant 9)  Letting high mood go… acceptance idea - “there is nothing I can do”. |
|  | Distract self (n = 1) | (Participant 2)  I distract myself |
|  | Being more present (n = 2) | (Participant 9)  Living in the present |
| 3.2. Changes in interpersonal behaviour (n = 8) | Observing others / seeing things from their point of view (n = 5) | (Participant 4)  It has helped me to understand that I need to understand people’s perspectives  (Participant 3) |
|  | Asking for what I need / asserting / negotiating (n= 2) | (Participant 3)  I am able to vocalise my mood more to others, as it develops: “I am getting this way, going to do X”. I am asking for what I need. |
|  | Knowing and understanding, but harder to put into practise / difficult to get techniques into the moment (n = 3) | (Participant 11)  I knew it and understood, but it’s harder to put into practice. |
|  | Sharing diagnosis with people I know (n = 1) | (Participant 10)  I felt more comfortable telling friends and family about attending. |
| 3.3. Impact on symptoms (n= 11) | Slowing thoughts down, calming the mind (n = 3) | (Participant 3)  My mind is slower and calmer. |
|  | General Calming (n=2) | (Participant 11)  I feel calmer sometimes |
|  | Symptoms persist (n=4) | (Participant 7)  Probably no impact. They have worsened over time but I don’t think this is because of the group. |
|  | Symptoms reduce (n=2) | (Participant 10)  At the start of the programme it also helped me to lift my mood out of a long period of feeling depressed. |
|  | Stabilise moods (n=4) | (Participant 11)  I found it useful, helpful to stabilise my moods. |
|  | Stop symptoms getting worse (n=4) | (Participant 10)  The therapy definitely helped me to keep high moods in check |
|  | Potential positive effects of drugs (n=2) | (Participant 8)  I can’t say for sure if it is just the therapy, or change in drugs (started new drugs 3-4 weeks before the start of therapy) |
|  | Not helpful when extremely high / low (n=1) | (Participant 1)  I’m sure it is helpful for a lot of people, but when manic and my brain was running I wasn’t in that place. |
| 3.4. Impact on relationships and functioning | Positive impact on others (n=3) | (Participant 8)  Calmer and easier on everyone |
|  | Building better relationships (n=2) | (Participant 5)  I am dealing slightly better with family and friendships. |
|  | Impact on work (n=4) | (Participant 5)  I’m getting back into the flow of work, I have increased my hours. |
|  | Reduced use of medication (n=1) | (Participant 9)  I initially relied less on meds and altered the doses – I made the decision to use the group instead |
