# Supplementary material for: Psychological therapy for mood instability within bipolar spectrum disorder: a single-arm feasibility study of a dialectical behaviour therapy-informed approach
Source: Pilot Feasibility Stud. 2020 Apr 15;6:46. doi: 10.1186/s40814-020-00586-1 (PMC7158125; doi:10.1186/s40814-020-00586-1)
Supplement: Supplementary file 2 — Additional file 2. Supplementary Material 2. Summary of Findings in Relation to Feasibility Aims. Table study findings in relation to each feasibility aim. [file 40814_2020_586_MOESM2_ESM.docx]

Supplementary Material 2: Summary of Findings in Relation to Feasibility Aims

| **Aim** | **Method of measurement** | **Outcome** | **Interpretation** |
| --- | --- | --- | --- |
| Acceptability of the intervention to patients | i) Proportion of participants completing treatment (defined as attending at least 50% of the 16 group therapy sessions);  ii) participant ratings of treatment satisfaction;  iii) qualitative analysis of semi-structured interviews with participants at the end of therapy;  iv) clinician satisfaction ratings;  v) qualitative analysis of semi-structured interviews with clinicians | i) 9/12 75% completed treatment  ii) 9/10 mostly or very satisfied overall post treatment; areas of higher and lower satisfaction  iii) generally acceptable with some suggested improvements  iv) all moderately or very satisfied,  v) overall viewed positively, with some specific recommendations | Broadly acceptable, some specific aspects that can be improved. |
| Acceptability of study procedures | i) Qualitative analysis of participant interviews;  ii) rates of completion of research measures. | i) no specific negative views of study procedures expressed other than for smartphone app;  ii) 10/12 participants completed post treatment and follow-up measures. | Procedures broadly acceptable; smartphone app requires improvement. |
| Feasibility of study procedures | i) Recruitment rate;  ii) Qualitative analysis of interviews with clinicians. | i) 14 ppts over 6 months  ii) viewed as possibly more suitable / feasible for certain patients. | Future feasibility RCT should be able to recruit at 2 participants per month using current referral routes, and could pilot additional referral routes. |
| Intervention having potential as a safe and effective approach for this client group | i) Reliable change in symptoms  ii) increase in suicidality;  iii) incidence of serious adverse events resulting from trial involvement. | i) no evidence for consistent reliable deterioration, at least half showed reliable improvements on measures of depression, anxiety, psychiatric symptomatology, recovery and quality of life; little evidence for reliable improvement in mania symptoms or on the ISS (symptoms in past 24 hours).  ii) no participants showed increase in suicidality.  iii) no serious adverse events resulting from trial. | Consistent with the potential to be a safe and effective intervention. |
| Performance of candidate outcome measures | Potential sensitivity to change | BDI-II, PHQ9, GAD7, CORE, BDRQ, QoL-BD showed reliable change; little change in mania measures (BMRS, ASRM) and ISS. | Majority of measures appear sensitive to change; unclear if absence of change on mania measures is due to insensitivity or floor effects. |
