# Supplementary material for: Psychological therapy for mood instability within bipolar spectrum disorder: a single-arm feasibility study of a dialectical behaviour therapy-informed approach
Source: Pilot Feasibility Stud. 2020 Apr 15;6:46. doi: 10.1186/s40814-020-00586-1 (PMC7158125; doi:10.1186/s40814-020-00586-1)
Supplement: Supplementary file 3 — Additional file 3. Protocol. Group Dialectical Behavioural Therapy for Mood Instability within Bipolar Disorder: An Open Trial. Study Protocol. Study protocol. [file 40814_2020_586_MOESM3_ESM.docx]

**Group Dialectical Behavioural Therapy for Mood Instability within Bipolar Disorder: An Open Trial**

**Study Protocol**

**Summary**

Background

Bipolar Disorders (BD), including all types, affects around 4-5% of the population. BD typically follow a relapsing course and are associated with considerable economic burden, estimated in 2002 at £2 billion annually in the UK (Das Gupta & Guest, 2002). BD are also associated with high personal and social costs, including a suicide rate that has been conservatively estimated at 9 times that of the general population (Dutta et al., 2007), and exceeds that associated with unipolar depression. Medication is generally considered to be the first line of treatment for individuals with BD (NIHCE, 2006) however a substantial number of individuals with BD continue to relapse despite use of medication (Gitlin et al., 1995). Furthermore, ongoing mood instability outside of full episodes is a significant difficulty experienced by many individuals with BD. This can range from residual mood symptoms between episodes of Bipolar I Disorder (which appear to affect over half of those with BD-I: Keitner et al., 1996) to the persistent short-lived swings in mood that characterise cyclothymia. As well as being distressing and destabilising, there is evidence that residual symptoms predict a worse course of BD (Benazzi, 2001; Judd et al., 2008) whilst those with cyclothymia have a greater likelihood of developing Bipolar I or II Disorder (Alloy et al., 2012).

Whilst there is evidence to suggest that certain psychological therapies (such as Cognitive Behavioural Therapy and group Psychoeducation) can be helpful in reducing risk of relapse and / or reduce symptoms of acute and residual bipolar depression, there are no evidence-based psychological therapies available for individuals experiencing ongoing mood instability, defined as either frequent sub-clinical shifts in mood outside of full episodes, or cyclothymic mood swings.

Dialectical Behaviour Therapy (DBT) was developed by Marsha Linehan as an approach for individuals with Borderline Personality Disorder (Linehan, 1993) and is recommended in the NICE clinical guideline for Borderline Personality Disorder (Department of Health, 2009). Since the initial development of DBT, studies have found it to be efficacious in other patient groups including individuals with substance use problems and those with treatment resistant depression. DBT aims to give individuals who experience rapid and intense shifts in affect skills for managing this, both in terms of their responses to shifts in affect, and in terms of shaping their environments in ways that promote emotional stability. DBT aims to help the client both to accept the realities of their situation and responses, and to make changes where it would be helpful. Traditional DBT consists of group-based skills training alongside individual therapy sessions, with therapist contact calls allowing generalisation of the skills learned into the client’s daily life.

Bipolar Disorders and Borderline Personality Disorder, whilst differing in a number of ways, share some common hypothesised underpinning processes, including elevated reward sensitivity and difficulties in emotion regulation. In addition, both disorders often involve rapid and extreme changes in affect. Despite this phenomenological and theoretical overlap there has little research evaluating DBT for BD. An open trial and a pilot randomised controlled trial have evaluated individual and family-based skills training for adolescents with BD (Goldstein et al., 2014; Goldstein, Axelson, Birmaher & Brent, 2007), whilst one pilot randomised controlled trial has investigated group-based DBT skills training for adults with BD (Van Dijk, Jeffrey & Katz, 2013). No studies to date have specifically investigated this approach as a means to help individuals with ongoing bipolar mood instability; rather, studies to date have included participants on the basis that they experience episodes of BD, regardless of whether or not their mood is stable between episodes. In the absence of evidence based psychological therapies for this group, DBT would appear to be a credible approach both in terms of target symptomatology and underpinning theory, and one which can be delivered in a group format reducing costs and potentially combatting social isolation and internalised stigmatisation.

We have developed a protocol for group-based DBT training that draws upon theoretical and empirical work into basic processes in Bipolar Disorder to adapt standard DBT for this client group. The format of the group is designed to make this intervention feasible to deliver within outpatient primary or secondary mental health settings whilst allowing for individualisation and generalisation of skills through individual skills-coaching sessions and the use of a mobile phone application (Rizvi et al., 2011).

Study Aims and Hypotheses

The aim of this study is to formally evaluate group DBT skills training for individuals with ongoing bipolar mood instability (frequent mood swings that persist outside of – or instead of - full episodes of illness). This will provide information that can be used to further refine the content and delivery of the intervention and inform the design of a pilot randomised controlled trial of the intervention.

Evaluation will primarily focus upon:

1. The feasibility and acceptability to participants and referring clinicians of i) the content of the intervention; ii) the delivery of the intervention (in terms of group and individual components, the “open” nature of the group element, and the use of experience sampling to support therapy).
2. Testing key outcome variables observed in participants in the group.
3. Evidence for mechanisms of change within participants in the group.

Hypotheses:

1. The intervention will be both feasible and acceptable to participants, clinicians and refers as measured through uptake and adherence rates, and qualitative feedback.
2. Between the baseline assessment and the end of the group participants will show i) a trend of decreasing levels of depressive and manic symptoms; ii) decreases in variability of symptoms and day-to-day mood; iii) increases in quality of life and sense of personal recovery.
3. The above changes will be i) associated with; ii) preceded by increases in mindfulness and group fit, and decreases in behavioural avoidance, positive and negative urgency, impulsivity and problematic beliefs about extreme mood states.

Experimental Design and Methods

**Design**

Open (uncontrolled) trial.

**Method**

*Participants*

Participants will be up to 16 individuals recruited through the Mental Health Assessment Team in the Exeter and East Devon area, and in the Mid Devon area if necessary.

In addition we will invite at least four members of staff from referring services to provide data on the acceptability of the Thrive programme.

Inclusion criteria for patient participants are: i) aged 18 or over; i) meets DSM-V criteria for Bipolar Spectrum Disorder; iii) current bipolar mood instability (over the past two years numerous periods with hypomanic symptoms that do not meet criteria for a hypomanic episode and numerous periods with depressive symptoms that do not meet criteria for a depressive episode, continuing into the past month); iv) client wishes to engage in psychological therapy that focusses primarily on ongoing mood instability and its consequences.

Exclusion criteria are: i) current substance dependence disorder; ii) currently receiving other psychological therapy; iii) the patient is currently actively suicidal; iv) presenting difficulties that are characteristic of Borderline Personality Disorder rather than Bipolar Disorder and that would be an early treatment target in standard DBT (frequent and serious deliberate self-harm, marked disturbance in ability to form or maintain interpersonal relationships); v) presence of another area of difficulty that the therapist and client believe should be the primary focus of intervention (for example panic disorder with agoraphobia, Post Traumatic Stress Disorder); vi) the information available suggests that the person may present a significant risk to other group members (such as aggression, likelihood of carrying out sexual or other exploitation); vii) the person lacks capacity to consent to treatment or research participation; viii) currently experiencing an episode of mania.

Inclusion criteria for staff participants are: i) mental health professional working within a service that can refer patients to the study.

*Materials*

Structured Clinical Interview for DSM-IV (SCID V: First, Williams, Karg & Spitzer, 2015): This is a clinician rated semi-structured interview for ascertaining diagnostic status according to the Diagnostic and Statistical Manual for Mental Disorders (DSM-IV: American Psychiatric Association, 2000) and is the gold standard for obtaining a diagnosis for research purposes.

*Outcome Measures*

Bech Mania Rating Scale (BMRS: Bech, Rafaelson, Kramp & Bolwig, 1978): An 11 item observer rated instrument, with higher scores corresponding to higher levels of hypomania or mania. This scale has been shown to have good internal consistency and construct validity.

Altman Scale for Rating Mania (ASRM: Altman, Hedeker, Peterson & Davis, 1997): A five item self-report measure of current manic symptom level that has been validated in patient populations .

The Bipolar Disorder Recovery Questionnaire (BDRQ: Jones, Mulligan, Higginson, Dunn & Morrison, 2013): A 36 item self-report measure that has been developed to measure personal recovery in people with Bipolar Disorder, as distinct from symptomatic or medical recovery. The BDRQ has been found to have adequate internal consistency and convergent validity.

The Brief Quality of Life in Bipolar Disorder scale (QoL.BD: Michalak & Murray, 2010): This is a 12 item self-report measure of quality of life that has been specifically designed for and validated with individuals with Bipolar Disorder.

Acceptability Questionnaires: Before starting the programme, participants will be asked about the extent to which they find it acceptable, and their general views on being offered a group treatment, as will referring clinicians. After completing the group participants and clinicians will be asked about their experience of the programme. The questionnaires contain both qualitative and quantitative elements, and includes questions about the smartphone app, based on a measure used in a previous published study (Rizvi et al., 2011).

Experience Sampling Measure (ESM):

Participants in the group will make use of a mobile phone application (“app”) which has been purpose designed by the experimenter for use in this study. This application alerts the individual 5 times per day over a period of 7 days. On each occasion the user is asked to rate his / her mood on a scale of -10 to +10. They are also asked if they have done anything since the last alert deliberately to regulate their mood, and if so, the extent to which this was helpful. This app will be used at the start and the end of the study to measure within and between day fluctuations in mood, and use of helpful coping responses.

During the group itself participants will continue to use the app, however during this phase it will be modified in order that it can be used therapeutically. Participants are asked to specify a “cut-off” at either end of the mood scale, and can determine the number of alerts they wish to receive per day. When the alert sounds they are asked to rate their mood as previously. If they rate themselves above their upper threshold (or below their lower one) they will be informed of this by the app and will be presented with recommendations (typically these will be skills learned in the group: participants can select their own recommendations in advance and programme them into the app). This means of using the app is intended to enhance the therapeutic efficacy of the group by helping participants to generalise the skills learned into everyday life at the times they are most needed. Participants will be able either to run the app on their own smartphone (if this is an android device) or will be able to borrow a device from the researchers (this will not be connected to the internet or be able to make calls). Data is returned to researchers either through downloading to the researchers’ PC directly from the device (if it is a University-owned device), or by the application sending anonymous data by email to the researchers’ server (if it is the participant’s own device).

*Process Measures:*

The UPPS-P Impulsive Behaviour Scale (Whiteside, Lynam, Miller & Reynold, 2005): A 59 item self-report measure that assesses five subscales (urgency, premeditation, perseverance, sensation seeking, and positive urgency) that are used to measure five distinct dimensions of impulse behaviour. This measure is included as a core proposed mechanism of action for DBT is via reduction of impulsive response to emotion.

The Behavioural Avoidance in Depression Scale (BADS: Kanter, Mulick, Busch, Berlin & Martell, 2007): A 25 item self report measure of avoidance behaviour and the impact of this upon social and daily functioning. This measure is included as our adaptations to the standard DBT protocol include an increased focus upon behavioural responses to emotion that exacerbate depression, specifically avoidance behaviours.

Kentucky Inventory of Mindfulness Skills (KIMS: Baer, Smith & Allen, 2004): A 39-item self-report inventory covering four facets of mindfulness: observing, describing, acting with awareness, and accepting without judgment. The KIMS is included as increase in mindful awareness and action are key hypothesised mechanisms by which DBT brings about more adaptive responses to ones emotions.

Group Fit Item: This asks participants to rate their sense of “fit” with the group on a single-item pictorial scale. This will be used to investigate whether there may be a relationship between a patient’s sense of fit with the group and his or her outcomes, as evidence suggests that social identification with treatment group may predict reduction of depression symptoms in individuals with Unipolar Depression (Cruwys et al., 2014).

Brief Hypomanic Attitudes and Positive Predictions Inventory (Brief-HAPPI: Mansell & Jones, 2006). A 30 item self report measure that measures problematic beliefs about mood states bipolar mood states. Scores on the Brief-HAPPI have been found to be elevated amongst individuals with Bipolar Disorder; it is included as such beliefs have been hypothesised to potentiate unhelpful responses to extreme mood states in bipolar disorder (Mansell et al., 2006), and the current study provides an opportunity to explore whether this variable is associated with change in impulsive responses to mood and symptom change over the course of treatment.

Inventory of Interpersonal Problems – Short Version (IIP-32: Barkham, Hardy & Startup, 1996). A 32 item self report measure of interpersonal difficulties across eight domains. This short version has been found to have comparable psychometric properties to the established 127 item version, but places a lower burden on participants in terms of time to complete. This measure is included as interpersonal effectiveness is a key target of DBT.

*Intervention*

The intervention consists of 16 sessions of group skills training based upon DBT, supported by individual meetings of up to 30 minutes that occur approximately monthly for each person, immediately following the end of each group session. The original DBT protocol and materials have been adapted for use with individuals with Bipolar Disorder by the applicants, and have been used clinically within two previous groups. Feedback from clinicians and patients involved in these groups has been used to iteratively refine the protocol and materials. The current version of the group follows a modular format: participants complete these modules in the following order (mindfulness, emotion regulation, mindfulness, distress tolerance, mindfulness, interpersonal effectiveness, consolidation), however if a participant leaves the group early, another participant can take his / her place at the start of the next module. This helps to maximise the number of people that can take part in the group. We will run two cycles of the entire 16 week group, to allow us to adequately test the feasibility of an open group design (whereby some participants may have started midway through the first cycle of the group, thus require the opportunity to complete the modules they missed, during a second cycle). No new participants will be able to join the group later than the start of the “emotion regulation” module of the second cycle, to ensure that all participants have the opportunity to be exposed to all modules. The group will finish after the end of the second cycle, or after the last participant has completed all modules, whichever is sooner.

After the end of the first group, “reunions” will be held every 3 months until all participants in both groups have had the opportunity to attend at least one reunion. All participants who have finished treatment will be invited to these, and sessions will focus upon celebrating practice done by participants and gains made, and by supporting participants with recently difficulties through group consideration of how skills learned could be applied.

*Procedure*

Patient participants will be identified by staff members in the xxxxxxx Mental Health Assessment Team (with the xxxxxxx Assessment Team being a second possible recruitment source if necessary) on receipt of referrals of individuals with Bipolar Disorder. If necessary we will also recruit from Recovery services (secondary mental health teams) in Exeter. Participants likely to meet study criteria will be informed about the study by the assessing staff member during a routine assessment appointment (given Participant Information Sheet). If necessary the patient and / or staff member can liaise with the research team to address questions about the study and study eligibility that arise at this stage. Patients expressing an interest in the study will then be referred to the AccEPT service, which hosts the project. Once referred, potential participants will be contacted to explain the study, have an opportunity to answer questions, and arrange an assessment appointment if they wish to proceed. At the assessment appointment the researcher will explain the study further, address questions and take informed consent. Following this the researcher will ascertain whether the participant meets study inclusion / exclusion criteria (the SCID will be used to investigate criteria pertaining to diagnosis). If the participant is not eligible for the study this will be explained and the participant referred onwards as appropriate. If the participant is eligible he / she will be invited to complete the “outcome” and “process” measures listed above, in addition to the standard assessment measures used within the AccEPT service. This can be done in the University or at home, in accordance with the participant’s preference. The participant will also be asked to complete the ESM recording period for a week prior to starting the group. He / she will be instructed in the use of the app at the end of the assessment session.

Participants will then undertake treatment as per the usual procedures at the AccEPT service; this involves completing measures of symptoms (ASRM and BDI-II) at each session. To this we will add the single item measure of group fit, and a single visual analogue scale of mood that day (from -10 to +10).

In addition participants will complete the following measures half way through the group cycle: UPPS-P, KIMS, BADS, HAPPI, IIP-32.

At the end of treatment participants will complete the standard set of “exit” questionnaires for the AccEPT service plus the outcome and process measures and SCID mood disorders section for the period since initial assessment, and will return for an individual meeting (standard practice).

Participants will be followed up six months after the end of treatment when they will complete the BDI-II, ASRM, QoL.BD, BDRQ, acceptability and group fit questionnaires, as well as the SCID mood disorders section for the past six months.

Staff participants will be identified by the CI informing the teams concerned at a team meeting. Information sheets and consent forms will be distributed. Staff will be able to opt to complete either an anonymous questionnaire, an interview, or both. Questionnaires will be left at the team base for staff members to complete, and a researcher will return at a later date to collect the completed anonymous forms. For staff wishing to take part in the interview, they will be asked to contact the research team to discuss further. If he / she remains interested an appointment will be arranged either in person (in the staff member’s place of work, or at the University of Exeter) or over the telephone. This will take around 20 minutes. Staff will be asked to complete these on two occasions: before the first participant starts the Thrive programme, and after the final patient completes.

*Statistical Analysis*

In order to assess acceptability and feasibility descriptive statistics will be used to represent participant uptake and retention across the study. In addition, acceptability interview data will be presented in terms of descriptive statistics, and qualitative data will be subjected to thematic analysis.

Mean change in questionnaire scores will be assessed through presentation of descriptive statistics and use of non-parametric tests (paired sample Wilcoxon Signed Rank Test).

In addition, if no data points are missing, the smart phone app should generate an approximate maximum of 560 measurements on participants’ mood [16 persons x 5 times per day x 7 days]. Within-day and between-day variations, as the temporal manipulation of process changes take place, will be of primary interest. The substantial number of data points will also provide a basis for modelling the data through repeated measure mixed method design, which will provide an average scenario of mood stability/variation after controlling for auto correlation that arise from repeated measures and controlling for time related variations. It is important to note that due to the small number of participants, generalizability from mixed model will be limited. Rather, the mixed method model is applied here to capture the real trend from repeated measures after controlling for auto correlations.

In addition, this study will generate rich data at the level of individual participants, and this will be explored using trend analysis to identify patterns of change across time, and possible predictors of therapy attrition and effect. However when it comes to reporting these data, care will be taken to balance the need for communicating potentially important findings with the need to protect participant anonymity, given that the report may be accessed by their peers in the group.

With regard to qualitative data written notes will be made of answers to interviews. These notes will be checked back with participants to ensure that they represent his / her own words. Thematic analysis will be used to identify key themes in the transcripts. This will be done separately by two researchers for the first five scripts. The themes identified by the two will be compared and a set of key emerging themes agreed. Additional transcripts will be coded by one researcher and contemporaneous notes made of the extent to which the existing themes are supported by the remaining transcripts, and of any new emergent themes. This work will be reviewed with the second researcher after a further 15 transcripts have been coded. The second researcher will compare the themes against the data and identify any that are redundant, inappropriate or have been missed. A final set of themes will be agreed by the two researchers.

Ethical Issues

**The intervention**

This study seeks to evaluate a well-established treatment, as applied to a population that differs in some ways from the main recipient population. There are three published studies reporting pilot trials of DBT with individuals with Bipolar Disorder (in adolescents and adults), all with favourable results (Goldstein et al., 2007, 2014; Van Dijk et al., 2013): our approach does not differ from these in essence, but includes some innovations particular to our research group (such as differentiating between types of positive mood and drawing upon laboratory research to inform strategies for downregulating excessive “high” mood states), and is tailored to a U.K. context. Furthermore, DBT is used internationally as part of routine clinical practice with individuals with Bipolar Disorder, according to clinical judgment. As such it has been previously used within our centre both on a group and an individual basis. Although numbers are small, service evaluation reveals a significant decrease in depression levels and a trend towards increased stability of depressive symptoms from the start to the end of the two groups conducted. Participants were not selected on the basis of mood instability, and were required not to be experiencing a full episode of illness, hence levels of manic symptoms in those patients treated were relatively low at baseline, making change on this variable unlikely to be apparent. Feedback from patients and clinicians has been used to improve the protocol. Potential ethical issues arising from delivery of the treatment will be approached as is standard within our service. Risk to / from self or others, if detected, will be responded to in accordance with our established protocols. All information provided by participants as part of their treatment will be treated in confidence and will be available only to members of the clinical team, other than where it is necessary to share information with other professionals as per standard practice. Participants will be informed of the circumstances under which confidentiality may be broken and will be consulted with as far as possible if this should become necessary. Treatment sessions will be recorded (if all members of the group agree to this). Recordings will not be shown to anyone outside of the clinical or research team unless all participants appearing consent that these can be used for training purposes. If they wish (as is standard in the AccEPT clinic) participants can opt to be contacted about future research conducted through our research centre.

Participants within group treatments delivered in our service develop a set of “ground rules” for the group (with the facilitators). These serve as a basis to address behaviour that may be detrimental to other group members. Group members are made aware that they do not have to divulge any information in the group that they do not wish to. The format of the group (which is described and set up as a course rather than a traditional therapeutic group) means that participants can engage with the material without necessarily having to share a great deal of personal information.

**Informed consent**

Before commencing treatment participants complete a consent to treatment form as part of our routine practice in the AccEPT clinic. They are sent this in advance and have time to consider it before their appointment and then to discuss it with the researcher / clinician. In addition participants will complete a study-specific participant information sheet (PIS) and consent form. Again they will receive this at least 2 days in advance and will have the opportunity to discuss it in the initial appointment. Participants judged not to have capacity due to severe symptom levels will be reassessed at a later point convenient to them and directed to sources of support in the meantime.

**Data Protection**

As per standard practice in the AccEPT service, information in the form of routine clinical notes and measures will be stored in individual patient files in locked filing cabinets in a locked office in the building that houses the clinic.

Additional information / measures gathered as part of this research study will be anonymised and stored in a locked filing cabinet in the locked office in the Department of Psychology, University of Exeter. Consent forms will be stored separately to data.

Information held on computer will be stored on the secure network hosted by the University of Exeter. Research information that is additional to that collected routinely by the clinic will be anonymised.

Staff members taking part in the study will be informed that they should not reveal detailed outcomes of individual clients (some of which may not be known to the research team), but rather comment upon their general impressions of the treatment. Patient participants will be informed that staff are being interviewed about the therapy, but will be asked about their impressions of this as a treatment option rather than individual patient outcomes.

**Use of the Smartphone Application**

The app will not store any identifying data, and data will be encrypted, preventing others with access to the mobile device from extracting meaningful information from the app. Data collected will be downloaded either directly by the researchers (for University-owned devices) or via email to the researchers’ server. There are no costs to the participant as they will only be asked to upload data where there is a WiFi connection. If participants use their own device the app will not interfere with its running.

Participants who borrow a device from the University will sign an agreement to return the device on completion of the study (a procedure that has been used previously within our research centre when lending out these devices). Researchers will offer to collect the device from the participant if this is more convenient. Participants will be given information to help them look after their device.

Participants will be informed on the information sheet and verbally that they should respond to the device alert only if it is safe to do so, just as they would if they received a standard message or phone call, and that they should switch the device off or to silent mode if its alerts will distract them or others in a way that places anyone at risk (e.g. when driving).

Mobile phone applications are beginning to be used within DBT by other research groups. A pilot study of use of such an application with individuals with substance use disorders found evidence to suggest that this was both acceptable and helpful (Rizvi et al., 2011).

Potential Benefits to Participants

Although there has not yet been a definitive randomised controlled trial of DBT for individuals with Bipolar Disorder, data from published pilot studies and from our service evaluation suggests that this can be a helpful approach. Indeed, we have delivered DBT for Bipolar Disorder previously because it was thought to be clinically indicated, particularly for individuals with rapid switches in mood for whom there is no current recommended psychological intervention. Therefore it is expected that most participants will experience at least some benefit from attending the group.

It is not expected that there will be any additional direct benefit from taking part in the research aspect of the study (completion of additional questionnaires) however the use of the smartphone application to support therapy is intended to be therapeutically beneficial.

Resources and Costs

The Chief Investigator is a Clinical Psychologist working within the AccEPT service, and has a small amount of time dedicated to the delivery of this project. The co-therapist will be a qualified Clinical Psychologist or Psychological Therapist. This individual will have half a day per week dedicated to co-running the group. As per our standard practice the group will be supported by an honorary assistant psychologist who will be responsible for setting up the group room, organising materials, administering session-by-session and process measures to participants, and storing and entering data. He / she will sit in on sessions and will operate a “clinic” at the end of the session in which he / she will help participants with use of the app (for example, inputting recommended responses to high / low mood). He / she will also download data from the participants’ devices (if necessary), or help them to submit their data, each week.

Any smartphone devices required will be provided from stock kept in the Department of Psychology, University of Exeter. The application has been constructed and will be supported by a specialist research computing programmer from the Wellcome Biomedical Hub at the University of Exeter.
